# Regulation of Transcription Termination of Small RNAs and by Small RNAs: Molecular Mechanisms and Biological Functions

**DOI:** 10.3389/fcimb.2019.00201

**Published:** 2019-06-12

**Authors:** Jiandong Chen, Teppei Morita, Susan Gottesman

**Affiliations:** ^1^Laboratory of Molecular Biology, Center for Cancer Research, National Cancer Institute, Bethesda, MD, United States; ^2^Faculty of Pharmaceutical Sciences, Suzuka University of Medical Sciences, Suzuka, Japan

**Keywords:** SgrS, ChiX, SraL, Hfq, CsrA, Rho

## Abstract

Accurate and efficient transcription termination is an important step for cells to generate functional RNA transcripts. In bacteria, two mechanisms are responsible for terminating transcription: intrinsic (Rho-independent) termination and Rho-dependent termination. Growing examples suggest that neither type of transcription termination is static, but instead are highly dynamic and regulated. Regulatory small RNAs (sRNAs) are key players in bacterial stress responses, are frequently expressed under specific growth conditions, and are predominantly terminated through the intrinsic termination mechanism. Once made, sRNAs can base-pair with mRNA targets and regulate mRNA translation and stability. Recent findings suggest that alterations in the efficiency of intrinsic termination for sRNAs under various growth conditions may affect the availability of a given sRNA and the ability of the sRNA to function properly. Moreover, alterations of mRNA structure, translation, and accessibility by sRNAs have the potential to impact the access of Rho factor to mRNAs and thus termination of the mRNA. Indeed, recent studies have revealed that some sRNAs can modulate target gene expression by stimulating or inhibiting Rho-dependent termination, thus expanding the regulatory power of bacterial sRNAs. Here we review the current knowledge on intrinsic termination of sRNAs and sRNA-mediated Rho-dependent termination of protein coding genes in bacteria.

## Bacterial sRNA Function and the Role of HFQ

Critical processes in bacteria, including those necessary for pathogenesis, are frequently regulated at multiple levels. Small regulatory RNAs play important roles in both bacteria and hosts. The bacterial small RNAs (sRNAs) function by base-pairing to target mRNAs, resulting in stimulation or inhibition of mRNA stability and translation. These sRNAs are usually around 100 nt long, and use a short seed region (8–15 nt) to form an RNA duplex with mRNA for regulation (Storz et al., 2011; Wagner and Romby, 2015). In *E. coli* and many related bacteria, sRNAs often act in concert with the RNA chaperone Hfq. Mutations in *hfq* are associated with attenuated virulence in many pathogens, suggesting critical roles for sRNAs in pathogenesis (Chao and Vogel, 2010), although, in a few instances, Hfq can act independently of sRNAs [see, for instance (Chen and Gottesman, 2017)].

Hfq, an Lsm/Sm family RNA binding protein, forms a ring-shaped homohexamer. Hfq binds to and stabilizes sRNAs and promotes their pairing with mRNA targets (Vogel and Luisi, 2011; Updegrove et al., 2016). Three different surfaces on the hexamer have been shown to be important for RNA binding. The proximal face binds polyU sequences, and mutations on this surface disrupt sRNA binding *in vitro* and sRNA stability *in vivo* (Otaka et al., 2011; Sauer and Weichenrieder, 2011). The distal face binds AAN repeats (Robinson et al., 2014), frequently found on mRNA targets of sRNAs. Some RNAs bind to the rim of Hfq through AU rich regions; the rim has also been implicated in helping bring sRNA and mRNA together (Panja et al., 2013). Most sRNAs are quite stable in the cell when bound to Hfq, but are presumably displaced from Hfq and degraded after pairing to mRNA targets (Massé et al., 2003; Schu et al., 2015). Thus, sRNA function and stability depend on the ability to bind Hfq properly.

Here we focus on sRNAs in *Escherichia coli*, one of the species in which these regulators have been well studied. sRNAs are usually expressed from dedicated and well-regulated promoters, but in some cases, sRNAs are processed from the 3′ end of mRNAs, and are thus dependent on the upstream mRNA promoters for expression (Miyakoshi et al., 2015b; Kavita et al., 2018). Regulation of sRNA promoters is often in response to specific cellular stresses, contributing to the cells' ability to adapt to changing environments. For instance, the iron-responsive RyhB sRNA is induced in response to iron deficiency and negatively regulates multiple iron binding proteins, thus helping the cell save iron for critical proteins (Massé and Gottesman, 2002). SgrS, on the other hand, is an sRNA made in response to accumulation of toxic sugar phosphates; it negatively regulates genes involved in the import of the sugar phosphates into the cell, while upregulating a phosphatase gene critical for detoxification of phosphosugar stress (Papenfort et al., 2013; Bobrovskyy and Vanderpool, 2014).

Regardless of whether an sRNA is processed from a longer transcript or not, a critical feature that enables sRNAs to bind Hfq is the presence of a Rho-independent terminator at its 3′ end (Otaka et al., 2011; Morita et al., 2017). This requirement suggests that transcription termination is crucial for both sRNA biogenesis and function. Recent work highlighting this process of sRNA termination, ways in which intrinsic terminators of sRNAs may be distinct from other intrinsic terminators, and the ways in which termination may be regulated are discussed in the first part of this review.

sRNAs, by binding to mRNAs, can affect many aspects of mRNA folding, translation, and decay, as well as access of RNA binding proteins. While sRNAs frequently act to alter ribosome binding and translation initiation, recent findings highlight how sRNAs can affect gene expression by blocking or facilitating premature Rho-dependent transcription termination within the genes of target mRNAs. We review the characteristics of these interactions in the second part of this review.

## Rho-independent Termination

Transcription termination that is independent of termination factors is known as Rho-independent termination, also called intrinsic termination [reviewed in Roberts (in press)]. Essential elements of a Rho-independent terminator consist of a GC-rich dyad repeat that forms a stem-loop (hairpin) structure followed by a T-rich stretch, generating a U-rich tail in the RNA after termination (Adhya and Gottesman, 1978). Rho-independent termination is achieved by formation of the stem-loop structure, which is facilitated by RNA polymerase pausing during transcription of the T-rich tract (Ray-Soni et al., 2016). The T-rich stretch is highly conserved among Rho-independent terminators in bacteria, while sequences of the stem-loop seem not to be conserved except for their GC-rich characteristic. Interestingly, T-rich stretches can be found in terminators for both eukaryotic RNA polymerase III and archaeal RNA polymerase (Arimbasseri et al., 2013; Maier and Marchfelder, 2019), suggesting that the intrinsic termination within a T-stretch is a fundamental characteristic of termination pathways. Although many of the T-rich stretches contain four to eight Ts, they are frequently disrupted with other nucleotides (d'Aubenton Carafa et al., 1990; Chen et al., 2013). Intrinsic termination can be directly measured *in vitro* by appearance of properly terminated transcripts, and *in vivo* by reporters to measure termination read-through [see, for instance (Morita et al., 2017)].

### Characteristics of sRNA Rho-Independent Terminators

**Figure 1:**
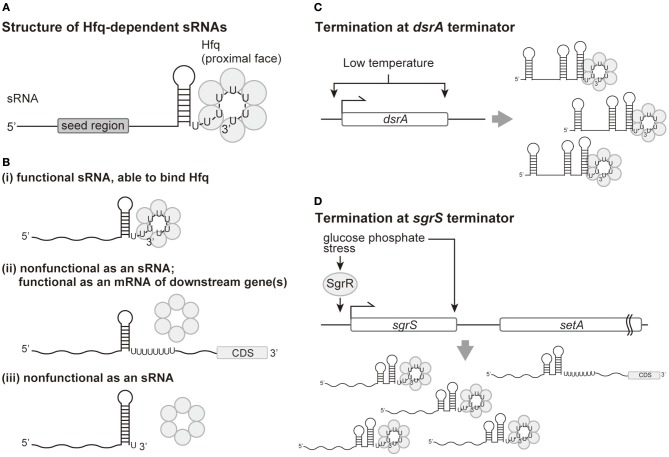
Production of the polyU tail of sRNAs by Rho-independent termination and the role of a polyU tail in Hfq binding and sRNA function. **(A)** Functional structure of an Hfq-dependent sRNA. The polyU tail of seven or longer is responsible for binding to the Hfq proximal face. The seed region is defined as the region which pairs with target mRNAs and is generally located 5′ to the terminator. **(B) (i)**: Termination at the seventh or longer position within the T stretch is necessary for functional sRNAs. **(ii)**: Transcripts with 3**′** extensions resulting from termination site read-through bind poorly to Hfq, resulting in non-functional sRNAs. **(iii)**: The transcripts with the U tail shortened by premature termination are no longer able to bind Hfq, resulting in non-functional sRNAs. **(C)** Low temperature increases both the level of transcription initiation and the termination efficiency for the gene encoding the DsrA sRNA. **(D)** Termination efficiency at the *sgrS* terminator is improved by glucose-phosphate stress, which also induces transcription initiation of *sgrS* by SgrR, a transcriptional regulator. Increased transcription termination will result in more production of SgrS and less expression of the downstream *setA* mRNA.

Transcription of genes encoding sRNAs are generally terminated by Rho-independent termination (Livny and Waldor, 2007). A notable feature of Rho-independent terminators of sRNAs is a consecutive T stretch longer than seven nucleotides, which is not necessarily found in all Rho-independent terminators (Otaka et al., 2011; Morita et al., 2017). The fact that discontinuous and relatively short T-rich stretches are found at many Rho-independent terminators implies that seven or more Ts found in sRNA terminators is not required for transcription termination. The transcribed long T stretch, i.e., a polyU tail of seven or more Us, is the primary element responsible for the binding of sRNAs to Hfq (Figure 1A). Studies of sRNA SgrS and others demonstrated that shortening the polyT stretch to four Ts in terminators of sRNA genes no longer produced functional sRNAs *in vivo* (Otaka et al., 2011). Additionally, the individual consecutive six uridines, including the last U, bind to the proximal face of the Hfq hexamer *in vitro* (Sauer and Weichenrieder, 2011). Consistent with these findings, deep sequencing analyses of Hfq-binding sRNAs verified that polyU tails of sRNAs longer than six Us are primary binding sites for Hfq *in vivo* (Holmqvist et al., 2016; Melamed et al., 2016) (Figure 1Bi). It should be noted that even shortening an *sgrS* variant from eight Ts to six Ts generated less functional SgrS, implying that six Us is not long enough to produce a fully functional sRNA (Otaka et al., 2011). Because, from a structural perspective, the six U tail seems to be sufficient for binding to Hfq, a question remains what the role of extra U(s) plays in sRNA function *in vivo*. In addition, some sRNAs possess a discontinuous U-rich tail disrupted with other nucleotides (Otaka et al., 2011; Morita et al., 2017). Effects of these discontinuities on the function and/or production of sRNAs also remain to be studied.

In contrast to a conserved polyT stretch, nucleotide sequences for the stem-loop structure seem not to be conserved between intrinsic terminators (Ishikawa et al., 2012; Morita et al., 2017). In fact, the SgrS sRNA was found to be fully functional with a heterologous terminator with a long polyU tail and hairpin stability approximately equal to that of the native terminator (Otaka et al., 2011). The stem-loop structure itself is critical for sRNA function, likely because without this structure termination efficiency is disrupted, resulting in 3′-extended transcripts. Nucleotide substitutions in the DsrA and RybB terminators that reduce thermodynamic stability of the stem were isolated as mutations affecting their function (Sledjeski and Gottesman, 1995; Balbontín et al., 2010). Consistent with the structure of Hfq specifically binding to the hydroxyl group at the 3′-end of the RNA *in vitro* (Sauer and Weichenrieder, 2011), a recent study found that read-through products of SgrS and RyhB with a polyU tract internal to the transcript did not bind Hfq *in vivo* (Morita et al., 2015). These findings suggest that 3′-extended transcripts resulting from read-through no longer function as sRNAs (Figure 1Bii). One sRNA, DicF, was reported to be produced both via termination and via 3′-processing by RNase III from a longer read-through transcript (Faubladier et al., 1990). However, a *dicF* coding region expressed from a plasmid and lacking the downstream processing site still generated functional DicF *in vivo* (Balasubramanian et al., 2016), suggesting that the intrinsic termination pathway is sufficient for the production of DicF, as with other sRNAs.

Another important feature of the terminator stem-loop structure is the strength of the stem, which affects the position of termination. A moderate but not too strong stem is needed to generate a polyU tail of seven nucleotides or longer. Extension of SgrS and RyhB terminator stems with additional G-C pairs resulted in premature termination and the generation of non-functional transcripts with polyU tails shorter than six (Morita et al., 2017; Figure 1Biii).

Based on the sRNAs studied thus far, the intrinsic terminators of sRNAs are expected to have unique features, forming a subset of the Rho-independent terminators in *E. coli* and possibly other bacteria. These features—a polyT stretch of seven or more and a moderate-strength stem-loop that enables termination after a stretch of seven or more Ts—can help in identifying potential Hfq-dependent sRNAs from genomic sequence analysis, and possibly help in distinguishing RNA transcripts that encode short proteins from those that have the potential to act as sRNAs. This would also be useful for the design and engineering of synthetic sRNAs for the control of specific gene expression. The absence of the critical characteristics for Hfq binding in the intrinsic terminators for mRNAs might prevent these mRNAs from binding to and blocking the proximal face of Hfq, where sRNAs must bind.

### Regulation of Rho-Independent Termination of sRNAs

A growing number of studies have found that sRNAs can be encoded upstream of ORFs, where the promoter upstream of the sRNA is responsible for expression of the downstream ORF. In these cases, expression of the ORF requires that transcription continue through the sRNA termination sequences, leading to a mRNA in which the embedded sRNA is non-functional. SgrS is co-transcribed with the downstream gene *setA* encoding a SET (sugar efflux transporter) family protein, although SgrS requires transcription termination at its own terminator for function (Sun and Vanderpool, 2011; Morita et al., 2015). SroC, one of the 3′ derived Hfq-binding sRNAs, is encoded within the *gltIJKL* operon encoding a Glu/Asp ABC transporter and likely needs to terminate at its own Rho-independent terminator for function (Vogel et al., 2003; Miyakoshi et al., 2015a). Given that Rho-independent termination impacts functional sRNA production, the downstream genes are assumed to be expressed discordantly from these sRNAs. Although the biological significance of the discordant expression of sRNAs and the downstream genes remains to be determined, a recent study revealed that such discordant expression by internal Rho-independent termination occurs frequently within operons and contributes to preferred expression levels for individual proteins (Lalanne et al., 2018).

Intriguingly, growing evidence suggests that the efficiency of transcription termination at Rho-independent terminators of sRNAs can be regulated by the same physiological and/or stress signals that induce initiation of sRNA transcription. Termination efficiency at the terminator for the DsrA sRNA, a positive regulator of RpoS translation, is increased at low temperature, where the *dsrA* promoter is most active (Sledjeski et al., 1996; Figure 1C). Similarly, an increase in termination efficiency at the SgrS and RyhB terminators was observed under conditions of both the cognate stress for transcriptional induction of these sRNAs and non-cognate stresses (Morita et al., 2015; Figure 1D). One can envision that this increase in termination efficiency would result in more effective production of sRNAs under specific conditions.

Previous studies on Rho-independent termination provide clues to the molecular mechanism by which Rho-independent termination is regulated. Lower levels of UTP nucleotide were found to improve transcription termination at several Rho-independent terminators *in vitro* (Farnham et al., 1982; McDowell et al., 1994). The density of RNA polymerase on an mRNA was found to influence the rate of transcription elongation/termination at Rho-independent terminators *in vivo* and *in vitro*, with higher transcription (more RNA polymerases) decreasing pausing and termination (Jacquet and Reiss, 1992; Epshtein and Nudler, 2003). Therefore, this linkage between promoter strength (transcription initiation) and termination provides a way in which stresses that decrease overall transcription activity on a given gene may result in increases in the termination efficiency. In addition, specific protein factors can be involved in regulation of Rho-independent termination. NusA is an essential protein which affects termination efficiency at Rho-independent terminators in multiple ways, by contributing to RNA polymerase pausing and by helping form and stabilizing RNA structures upon termination (Nudler and Gottesman, 2002; Guo et al., 2018; Holmqvist and Vogel, 2018), and thus could regulate termination in response to stresses. Hfq can be excluded as a candidate factor because it seems not to be involved in the termination at sRNA terminators (Morita et al., 2015).

Most *in vivo* experiments on Rho-independent termination have been carried out with protein-coding genes as templates. The situation at the sRNA terminators might be different from that at the mRNA terminators because the protein-coding genes are typically longer than sRNA genes. For instance, for short sRNA-encoding transcripts, dissociation of the sigma factor from the RNA polymerase, usually assumed to occur soon after transcription initiation, might not occur before the polymerase reaches the intrinsic terminator, possibly changing the efficiency of termination. A recent long-read RNA sequencing strategy enabled analysis of intact transcripts and revealed that the degree of readthrough of several mRNA terminators also varied between growth conditions (Yan et al., 2018). A critical question for future investigation will be how Rho-independent terminators are modulated and whether these mechanisms of modulation are specific to sRNA terminators, or also affect those for mRNAs.

## Rho-dependent Transcription Termination

In *E. coli*, 20–30% of transcription events are terminated in a Rho-dependent manner [(Peters et al., 2009, 2012); reviewed in Roberts (in press)]. While Rho is generally not used to form the 3′ ends of regulatory sRNAs, its ability to terminate mRNAs can be regulated by sRNAs.

Transcription termination factor Rho is an ATP-dependent RNA helicase/translocase, which can bind to a sequence motif called the Rho utilization (*rut*) site on the nascent transcript, translocate along the naked RNA and dissociate the elongation complex to terminate transcription (Grylak-Mielnicka et al., 2016; Ray-Soni et al., 2016). A typical *rut* site is a single-stranded, ribosome-free, cytosine-rich/low-guanine RNA sequence with a length of ~60–80 nucleotides. Thus, molecular or cellular processes that change ribosome occupancy on mRNA or the single-stranded nature of an RNA stretch at or adjacent to the *rut* site may regulate Rho's function. Rho forms a homohexamer ring, and uses its N-terminal OB-like protein fold to bind cytosine-rich sequences, while a region near the C-terminal part of the protein binds to the RNA threaded in the channel; the latter binding event activates Rho's ATPase activity, driving its translocation (Skordalakes and Berger, 2006). Several models of Rho-dependent transcription termination have been proposed (Peters et al., 2011; Ray-Soni et al., 2016), but the molecular details in terms of the mechanism of Rho translocation and RNA polymerase dissociation on Rho-dependent terminators remain to be fully determined. *In vitro* approaches to study Rho-dependent termination and its regulation by sRNAs have been recently described (Nadiras et al., 2018). *In vivo* approaches primarily depend on defining Rho-dependent termination with Rho-specific inhibitors such as bicyclomycin, and evaluating effects of sRNAs on mRNA expression by measuring read-through products with reporter assays, quantitative PCR or deep sequencing (Hussein et al., 2015; Elgamal et al., 2016; Sedlyarova et al., 2016).

### Rho-Dependent Transcription Termination Affected by sRNAs and RNA-Binding Proteins

Rho-dependent termination at the 3′ end of protein-coding genes is well documented, and this process is not believed to be significantly regulated. Rho can also terminate transcription inside genes and within operons, and this is likely a highly regulated process given the fact that the translation status of mRNAs can directly affect Rho access to *rut* sites inside coding sequences (Adhya and Gottesman, 1978). Genome-wide analysis of RNA Polymerase redistribution in the presence of the Rho-specific inhibitor bicyclomycin (BCM) found that Rho terminates transcription intragenically within ~100 genes (Peters et al., 2009, 2012). The observation of such intragenic termination sites raised the possibility that factors that can affect translation, such as sRNAs or RNA-binding proteins, may regulate Rho-dependent termination at these sites.

**Figure 2:**
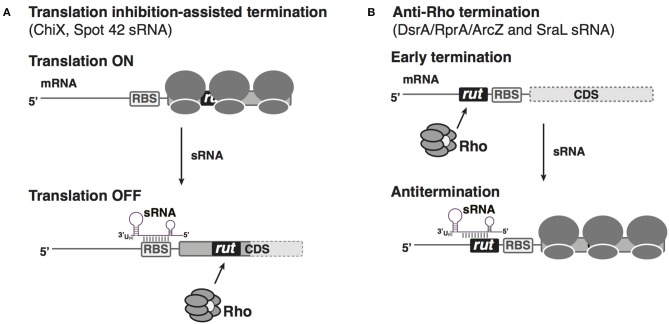
sRNA-based negative or positive regulation of Rho-dependent transcription attenuation in bacteria. **(A)** Translation inhibition by sRNA assists Rho-dependent termination. When the accumulation of *Salmonella* ChiX or *E. coli* Spot 42 sRNA is low, the mRNAs *chiPQ* and *galETKM* are translated well. When ChiX or Spot42 sRNA is produced at high levels, under specific environmental conditions, sRNA annealing blocks the ribosome binding site of the corresponding mRNA target (*chiP* or *galK*, respectively); reduced translation allows Rho loading onto a cryptic *rut* site to terminate transcription inside the genes (dotted pale portion of box represents untranscribed portion of the mRNA). **(B)** Antagonization of Rho-dependent attenuation by sRNAs. In genes with long 5′ UTRs, *rut* sites can allow Rho termination, blocking expression of the downstream gene (dotted box represents untranscribed coding region). sRNAs binding within the 5′ UTR can block Rho access to the *rut* sites, thus acting as anti-terminators for the downstream CDS.

A seminal study documented sRNA-mediated regulation of Rho-dependent termination in *Salmonella* (Bossi et al., 2012). ChiX is an Hfq-dependent sRNA that can bind to the *chiP* mRNA, encoding the outer membrane channel (porin) for chitosugars, at its ribosome binding site, inhibit translation and trigger *chiP* mRNA decay (Figueroa-Bossi et al., 2009). Interestingly, ChiX-mediated translation inhibition has a secondary effect. By blocking entry of ribosomes on *chiP* mRNA, ChiX binding exposes an intragenic *rut* site for Rho loading, leading to premature termination of transcription inside the *chiP* coding sequence (Figure 2A). This regulation not only reinforces the inhibition of *chiP* translation but also leads to a polarity effect, inhibiting expression of the downstream *chiQ* gene in the same operon (Bossi et al., 2012). When chitosugars are available and thus the transporter is needed, ChiX is destroyed by interaction with an RNA decoy, induced dependent on chitosugar sensing (Figueroa-Bossi et al., 2009; Overgaard et al., 2009). Therefore, regulation coordinates with other transcriptional circuits to ensure genes responsible for utilization of chitin-derived oligosaccharides are switched on only when chitosugars are present. In a second example, the *E. coli* Spot 42 sRNA base pairs with the *galK* leader, the third gene in the *galETKM* operon, resulting in translational inhibition of *galK* as well as an increased Rho-dependent transcription termination, possibly by interfering with translational coupling between ribosomes translating through *galT* with those initiating translation of *galK*, and thus allowing Rho access (Wang et al., 2015; Figure 2A). This type of regulation allows discoordinate expression of operon genes in response to metabolic needs of the cells.

In the cases described above, sRNAs facilitate Rho access to *rut* sites by interfering with mRNA translation, consistent with the consensus that mRNA sequences devoid of translating ribosomes are preferable targets for Rho binding and regulation. However, sRNAs can also positively regulate genes, raising the possibility that in some cases, sRNAs might antagonize Rho function by blocking Rho binding and termination. In addition to the 3′ end of genes and the intragenic regions containing *rut* sites, long 5′ UTRs (>80 nt) of mRNAs are another reservoir of potential sites for Rho-dependent termination. Transcriptomic analyses of RNA samples from *E. coli* treated with sublethal concentrations of Rho inhibitor BCM (Sedlyarova et al., 2016) suggested that, out of the 1,200 5′ UTRs longer than 80 nts, at least 250 were targets for Rho-dependent termination. One such 5′ UTR is the 567 nt leader of *rpoS*, an mRNA known to be positively regulated by sRNAs (Battesti et al., 2011). In-depth analysis of regulation in the *rpoS* 5′ UTR revealed that the three base-pairing sRNAs (RprA, DsrA, and ArcZ) that activate *rpoS* mRNA translation can antagonize Rho-dependent premature termination in the *rpoS* leader (Sedlyarova et al., 2016; Figure 2B). Such a regulatory mechanism is likely not limited to *rpoS*, but may apply to many other putative targets of these three sRNAs, as well as other positively regulated mRNAs. As optimal growth of *E. coli* in the stationary stress condition relies on expression of the stationary sigma factor RpoS, the sRNA-mediated upregulation of RpoS production both via increasing ribosome entry and via anti-Rho-dependent premature termination provides an important way for sRNAs to contribute to the response to stress.

In another example of an sRNA preventing Rho-dependent termination, the *Salmonella* SraL sRNA was reported to base-pair with the leader for *rho* mRNA, protecting it from transcription attenuation and thus increasing levels of full-length *rho* mRNA (Silva et al., 2019; Figure 2B). While the physiological role of this regulatory interaction is not known, it raises the possibility that under certain conditions the cell might modulate Rho levels and thus Rho-dependent termination via the SraL sRNA. For both the *rpoS* and *rho* leader, it is still not clear how sRNA binding prevents Rho-dependent premature termination. In both cases, the sRNA binds upstream of the RBS site, and in the absence of corresponding sRNAs, Rho can prematurely terminate transcription in the leader. Therefore, it is very likely that sRNA blocks Rho loading by occluding a *rut* site, either directly by overlapping the site, or indirectly via RNA structure rearrangements. The latter regulatory mechanism is exploited by the *E. coli* CsrA RNA-binding protein in regulating *pgaABCD* operon expression (Figueroa-Bossi et al., 2014). *pgaA* mRNA is prematurely terminated in its leader in a Rho-dependent manner, and this regulation relies on CsrA binding to a region in the long *pgaA* leader; CsrA binding refolds the *pgaA* leader, exposing an intragenic *rut* site for Rho binding to prematurely terminate transcription.

In addition to the role of Hfq in chaperoning sRNA stability and function, sRNA-independent roles of Hfq also have been reported (Wagner and Romby, 2015; Chen and Gottesman, 2017). Rabhi et al. (2011) elegantly demonstrated that Hfq, as a general RNA-binding protein, can antagonize Rho-dependent transcription termination at a prototypical terminator (λtR1) both *in vitro* and *in vivo*. The antitermination activity of Hfq depends on (1) its distal face binding to an A-rich sequence motif upstream of *rut* sites in λtR1, and (2) physical interaction of Hfq with Rho protein; this interaction can block Rho binding to other proteins, such as NusG, which has been shown to stimulate Rho termination at some terminators (Mitra et al., 2017). In this case, Hfq directly modulates Rho's RNA-DNA unwinding activity, rather than simply blocking access of Rho to RNA. Such a regulation is distinct from the roles that sRNAs and CsrA play in regulating Rho access to RNAs. Nonetheless, these reports of both negative and positive regulation of Rho-dependent termination by Hfq-dependent sRNAs or RNA-binding proteins reinforce the idea that RNA-based regulation of termination is widely exploited by bacteria.

### Autoregulation of Rho

Rho is an abundant protein in many bacteria, including *E. coli* (Grylak-Mielnicka et al., 2016). It is autoregulated through a transcription attenuation mechanism at its own leader in *E. coli* and in other bacteria (Barik et al., 1985; Matsumoto et al., 1986; Ingham et al., 1999), emphasizing the importance of cells maintaining an appropriate level of Rho and presumably of Rho-dependent termination. In *Rhodobacter capsulatus*, Rho is significantly induced under anaerobic growth (Jäger et al., 2004), suggesting a role of Rho in response to changing environments. The recent finding of an sRNA, SraL, that regulates Rho expression, described above (Silva et al., 2019), further highlights the likelihood that cells adjust termination under some conditions by adjusting Rho availability.

## Summary and Issues for the Future

sRNA research in the last 20 years have led to a growing appreciation of the ways in which small regulatory RNAs participate in stress circuits in bacteria. In the simplest terms, a sRNA will be induced in response to a stress, and it will pair and regulate the expression of mRNAs encoding proteins that help the cell deal with stress, and will thus contribute in significant ways to the recovery from the stress (Holmqvist and Wagner, 2017; Fröhlich and Gottesman, 2018). Here we have reviewed recent studies that highlight the complexity of this process and its intersection with transcription termination. In particular:

1. While transcription initiation remains the most important contributor to sRNA synthesis and accumulation, proper termination of the sRNA transcript is equally important for its function. The requirements for efficient termination and binding of the nascent sRNA to Hfq have likely selected for particular characteristics of intrinsic terminators for sRNA-encoding genes.
2. Recent work shows that the efficiency of proper intrinsic termination at sRNA terminators can be changed by cellular stress, by mechanisms still to be explored. It remains to be determined if these changes in termination affect intrinsic termination in general or are specific to sRNA terminators. For sRNAs, however, stress that changes the efficiency of termination will affect the fraction of transcripts that become functional sRNAs. This provides new insight into the mechanism of Rho-independent termination, which has been considered a simple, unregulated process.
3. Rho-dependent termination requires access of Rho to single-stranded RNA, and thus sRNAs that block translation may open up Rho entry sites within genes, reinforcing negative regulation of the mRNAs. sRNA binding to otherwise untranslated RNAs (for instance, in 5′ UTRs) can positively regulate downstream ORFs by blocking Rho entry.

The extent and importance of these levels of regulation are just beginning to be realized. Much still remains to be addressed to fully understand the effect of sRNAs on transcription termination. Unlike translational repression and mRNA degradation, effects on termination must occur rapidly, during the transcription process. This suggests that sRNA pairing with mRNAs must be happening co-transcriptionally; how often does that happen? Are there properties of the particular mRNAs, sRNAs, or transcriptional machinery that will favor this? In addition to the trans-acting sRNAs discussed here, the cis-regulatory riboswitches can also control gene expression through modulation of Rho-dependent transcription termination (Hollands et al., 2012; Proshkin et al., 2014; Bastet et al., 2018). It seems likely that, in addition to uncovering new levels of stress regulation, these studies may lead to a deeper understanding of transcription termination itself.

## Author Contributions

All authors contributed equally and directly to the preparation of this review, and all approved it for publication.

### Conflict of Interest Statement

The authors declare that the research was conducted in the absence of any commercial or financial relationships that could be construed as a potential conflict of interest.
